# Synthesis of zeolites Na-A and Na-X from tablet compressed and calcinated coal fly ash

**DOI:** 10.1098/rsos.170921

**Published:** 2017-10-18

**Authors:** Tao Hu, Wenyan Gao, Xin Liu, Yifu Zhang, Changgong Meng

**Affiliations:** School of Chemistry, Dalian University of Technology, Dalian 116024, People's Republic of China

**Keywords:** coal fly ash, Na-A, Na-X, hydrothermal synthesis, tablet compression

## Abstract

Zeolites Na-A and Na-X are important synthetic zeolites widely used for separation and adsorption in industry. It is of great significance to develop energy-efficient routines that can synthesize zeolites Na-A and Na-X from low-cost raw materials. Coal fly ash (CFA) is the major residue from the combustion of coal and biomass containing more than 85% SiO_2_ and Al_2_O_3_, which can readily replace the conventionally used sodium silicate and aluminate for zeolite synthesis. We used Na_2_CO_3_ to replace the expensive NaOH used for the calcination of CFA and showed that tablet compression can enhance the contact with Na_2_CO_3_ for the activation of CFA through calcination for the synthesis of zeolites Na-A and Na-X under mild conditions. We optimized the control variables for zeolite synthesis and showed that phase-pure zeolite Na-A can be synthesized with CFA at reactant molar ratio, hydrothermal reaction temperature and reaction time of 1.3Na_2_O: 0.6Al_2_O_3_: 1SiO_2_: 38H_2_O at 80°C for 6 h, respectively, while phase-pure zeolite Na-X can be synthesized at 2.2Na_2_O: 0.2Al_2_O_3_: 1SiO_2_: 88H_2_O at 100°C for 8 h, respectively. The composition, morphology, specific surface area, vibration spectrum and thermogravimetry of synthesized Na-A and Na-X were further characterized.

## 1. Introduction

Coal fly ash (CFA) is the major residue from coal and biomass combustion and is mainly composed of fine-grained particles of SiO_2_ and Al_2_O_3_. In 2015, more than 1 billion tons of CFA was produced worldwide [1]. The amount of CFA produced grows continuously, making the disposal and handling of CFA an important issue that strongly impacts various aspects of our daily life, especially the environment [2–4]. Therefore, it is important to develop techniques for the utilization of CFA and several have been proposed in this category [5–9]. CFA has been used directly for soil remediation [10–13], embankments or building [14–16], as adsorbent for waste and contaminant handling, gas separation and capture and mineral recovery [17–27], as catalyst or catalyst support [9,28], etc. Furthermore, it is of more economic significance to develop eco-friendly techniques that convert CFA to value-added products. CFA utilization as raw material for production of cement and glass has also been reported [29].

Most zeolites are periodic framework compounds of aluminium silicates formed by interconnected SiO_4_ and AlO_4_ tetrahedrons [30]. Owing to the crystalline nature and the abundant AlO_4_ sites, zeolites possess well-defined distribution of pore size and outstanding cation-exchange capacity, enabling extensive industrial applications as adsorbents [31–34], catalysts [35], catalyst supports [36,37] and cation-exchange materials [38–42]. Zeolites are conventionally synthesized with sodium silicate and aluminate through hydrothermal crystallization. CFA is mainly composed of particles of SiO_2_ and Al_2_O_3_ at micrometre dimension with a weight percentage up to 85 [8,43,44] and can principally be used as the precursor of Si and Al for zeolite synthesis [45–56]. However, SiO_2_ and Al_2_O_3_ are in the form of their most stable minerals in CFA, such as mullite and quartz. [8]. They must be chemically converted to reactive precursors by reactions with concentrated NaOH solution or calcination with solid NaOH or KOH at high temperature and the process is not energy efficient. Following the first synthesis of zeolite with CFA by Holler & Wrisching [57], the reported procedures for zeolite synthesis are all through hydrothermal crystallization using raw or NaOH- or KOH-calcinated CFA [5,58].

Developing cost-effective and eco-friendly routes for the synthesis of zeolite from CFA is of great economic and technological significance. Alternative energy sources, such as ultrasonic and microwave radiation, are proposed for the hydrothermal synthesis to lower the cost for zeolite synthesis with CFA, though they are less meaningful for industrial mass production [5,59]. Apart from these, it would be more interesting to replace the expensive NaOH and KOH used for the calcination and activation with low-cost alkalis, such as Na_2_CO_3_. However, due to the low alkalinity of Na_2_CO_3_ and the sluggish reactions at interface, higher calcination temperature will be required unless the process is intensified with other procedures. As the size of Al_2_O_3_ and SiO_2_ particles is of micrometre dimension, their reactions will, in principle, be greatly promoted by close contacts with Na_2_CO_3_ or NaOH. In this work, we used tablet compression to intensify the calcination of CFA with Na_2_CO_3_. We showed that the reactions between CFA and Na_2_CO_3_ are significantly promoted by the effective contacts resulting from the tablet compression and CFA can be fully converted into precursor for zeolite synthesis. We also showed phase-pure Na-A can be synthesized with calcinated CFA in low alkalinity solution under low temperature, while phase-pure Na-X can be synthesized in the same way but with the introduction of silica sol. The effects of varying Na_2_CO_3_/CFA ratio and reaction temperature were also investigated and the optimum conditions for the synthesis of Na-A and Na-X were determined.

## 2. Experimental

### 2.1. Material and methods

**Figure 1.**
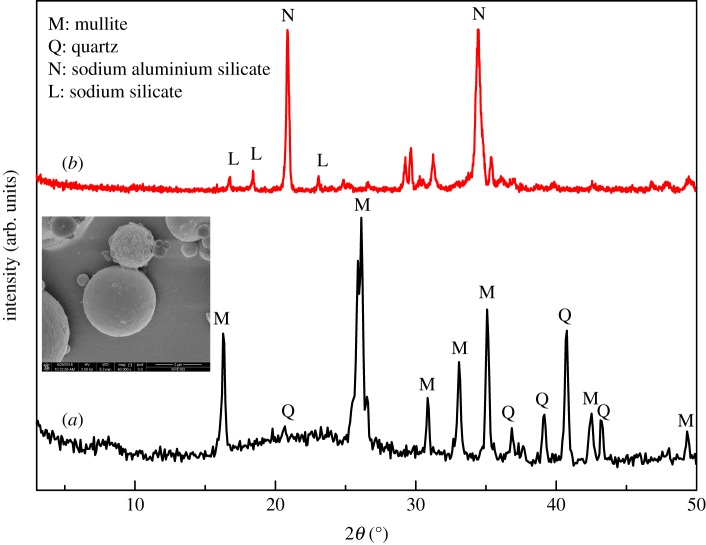
XRD patterns of the raw CFA sample (*a*) and the Na_2_CO_3_ and CFA mixture after being calcinated at 800°C for 2 h (*b*). The SEM image of raw CFA sample is shown as the inset of (*a*). (Mullite: PDF 83-1181; quartz: PDF 70-3755; sodium silicate: PDF 16-0818; sodium aluminium silicate: PDF 09-0463.)

CFA was obtained from Inner Mongolia, People's Republic of China. Chemical composition of the CFA sample was analysed with X-ray fluorescence (XRF) and the results are summarized in table 1. X-ray diffraction (XRD) was used to determine the crystalline phase in CFA and the morphology was determined with a scanning electron microscope (SEM). As shown in figure 1*a*, CFA sample is mainly composed of microspheres of quartz and mullite. The measured Si/Al molar ratio of 1.67 in the sample is suitable for the synthesis of Na-A zeolite. As the synthesis of Na-X zeolite requires higher Si/Al molar ratio, silica sol was introduced as additional Si source and sodium hydroxide was also added.

**Table 1.** The chemical composition of coal fly ash.

component	Al_2_O_3_	SiO_2_	Fe_2_O_3_	CaO	TiO_2_	P_2_O_5_	SO_3_	ZrO_2_	SrO	MgO
content (%)	45.48	44.86	2.73	2.69	2.19	0.45	0.35	0.30	0.25	0.22

### 2.2. Synthesis of zeolite Na-A and zeolite Na-X

Synthesis of Na-A zeolite was done by a two-step process. First, a tablet compression machine was used to treat the mechanically mixed CFA and Na_2_CO_3_. The resulting mixture was calcinated at 800°C for 2 h and then cooled to room temperature. According to the XRD patterns of the CFA sample before and after calcination (figure 1), the mullite and quartz phases in CFA disappear and are converted into sodium aluminium silicate (NaAlSiO_4_) and sodium silicate (Na_2_SiO_3_) after calcination at 800°C. It is hard to get phase-pure zeolite Na-A through the hydrothermal process if the mixture of CFA and Na_2_CO_3_ has not been tablet-compressed. The calcinated mixture was mixed with different quantity of deionized water at room temperature for 1 h under magnetic stirring, and then further heated to different temperatures and subjected to hydrothermal crystallization for a period of time. After that, the reaction mixtures were annealed to room temperature, filtered, washed with deionized water and then the solid residues were dried at 100°C for 24 h before further measurement and characterization. The addition of silica sol and sodium hydroxide to the mixture before stirring is needed to get phase-pure zeolite Na-X.

### 2.3. Characterization

The crystalline phase was identified by powder XRD (XRD-6000, Shimadzu, Japan) using CuKα radiation (40 kV and 100 mA). The scanning rate was 0.06° s^−1^, and 2*θ* range was 3–40°. The morphology of the as-synthesized materials was examined using a SEM (Nova NanoSEM450, FEI). The chemical compositions were determined by XRF (XRF-1800, Shimadzu, Japan). Fourier transform infrared (FT-IR) spectra were collected by a Nicole Avatar 360 FT-IR spectrometer (USA) over the range of 400–4000 cm^−1^ with a resolution of 2 cm^−1^ using the KBr disc technique. Thermogravimetric analysis (TGA) was also performed for the zeolitic products between 25°C and 900°C with a heating rate of 10°C min^−1^, with flowing air, using an SDT 2960 simultaneous DSC–TGA from TA Instruments. The Brunauer–Emmett–Teller (BET) surface area was calculated using nitrogen adsorption data at −196°C collected using a Micromeritics ASAP-2020 porosity analyser (USA). The pore volume, average diameter and specific surface area of zeolite samples were determined using the BET method and Novae, Quantachrome equipment.

## 3. Results and discussion

The reactant ratios of SiO_2_/Al_2_O_3_ and Na/SiO_2_ in the reaction mixture are crucial parameters to determine the crystallinity and properties of the zeolite products from hydrothermal synthesis. We have determined the composition of the CFA sample (table 1). As we used Na_2_CO_3_ instead of NaOH for the synthesis, we used Na_2_CO_3_/CFA mass ratio to describe the alkaline content in the reaction mixture for convenience. The effects of these parameters on the crystallinity and properties of zeolite synthesized from calcination-activated CFA were investigated in detail.

### 3.1. Hydrothermal synthesis of zeolite Na-A from coal fly ash

The impacts of Na_2_CO_3_/CFA mass ratio and crystallization temperature on the crystallinity of the resulting zeolite synthesized from calcinated CFA powder were investigated.

**Figure 2.**
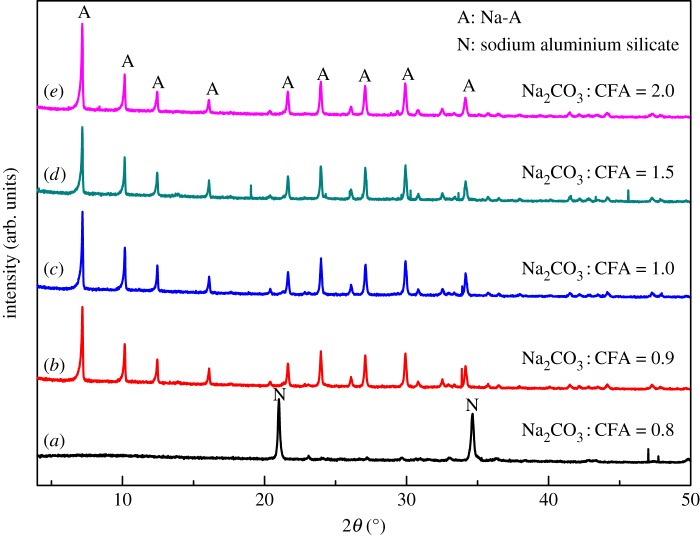
The effect of Na_2_CO_3_/CFA mass ratio on the structure of the products (Na-A: PDF 38-0241; sodium aluminium silicate: PDF 09-0463).

Controlled experiments were carried out, with Na_2_CO_3_/CFA mass ratio of 0.8, 0.9, 1.0, 1.5, 2.0, calcinated at 800°C for 2 h and hydrothermally treated at 100°C for 6 h, to investigate the influence of alkalinity on the formation of zeolite. Figure 2 shows the XRD patterns of products obtained at different Na_2_CO_3_/CFA mass ratios. According to the XRD pattern of calcinated Na_2_CO_3_/CFA mixture (figure 1), the product is mainly unconverted NaAlSiO_4_ formed during calcination with the lowest Na_2_CO_3_/CFA mass ratio of 0.8 (figure 2*a*). This is in good agreement with previous finding of Jiang *et al*. [30]. With the gradually increased Na_2_CO_3_/CFA mass ratio, the diffraction peaks of Na-A zeolite crystal appear and become stronger and sharper in the XRD patterns of the synthesized samples. After the Na_2_CO_3_/CFA mass ratio reaches 0.9 (figure 2*b*), the phase-pure Na-A zeolite with good crystallinity was obtained. When the Na_2_CO_3_/CFA mass ratio is further increased to 1.0–2.0, the phase-pure Na-A zeolite samples with good crystallinity are obtained. These results indicate that low Na_2_CO_3_/CFA mass ratio is not beneficial for the dissolution and incorporation of NaAlSiO_4_ into the Na-A zeolite framework. Within the inspected Na_2_CO_3_/CFA mass ratio range, high Na_2_CO_3_ content would promote the formation of zeolite and the optimal Na_2_CO_3_/CFA mass ratio for the preparation of Na-A zeolite is 1.0–2.0.

**Figure 3.**
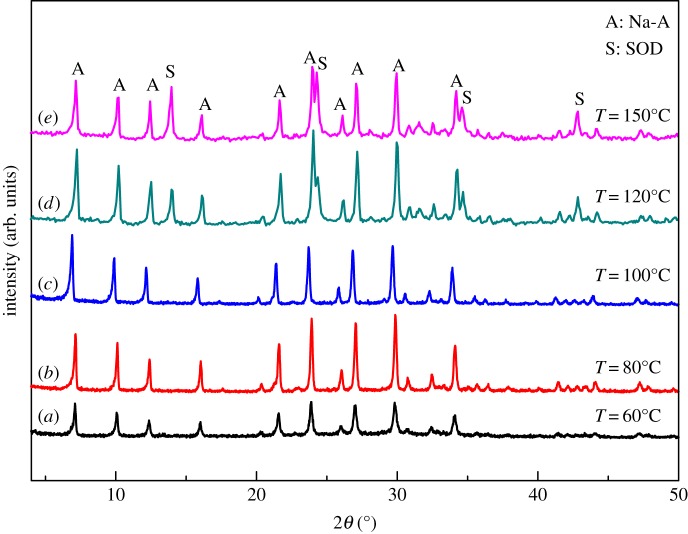
The impact of crystallization temperature on the structure of the products (Na-A: PDF 38-0241; SOD: PDF 037-0476).

We then moved on to investigate the effect of crystallization temperature on the crystallinity of the zeolite product. Controlled experiments were carried out from 60°C to 150°C with the other conditions unchanged. The XRD patterns of the samples obtained after crystallization at different temperatures for 8 h are shown in figure 3. The characteristic XRD peaks of Na-A zeolite appear even after crystallization at 60°C. However, these peaks are broad and short, showing the low crystallinity and purity of the product (figure 3*a*). The XRD peaks are narrowed and sharp from 60°C to 100°C (figure 3*a–c*), showing that in this temperature range, rising temperature would promote the crystallization of Na-A and there is a positive correlation between the crystallinity of the zeolite product and the crystallization temperature. According to the XRD pattern, phase-pure Na-A can be obtained from 60°C to 100°C. As zeolite Na-A is thermodynamically less stable than sodalite (SOD) in the current reaction mixture, we noted the SOD phase from 120°C to 150°C (figure 3*c–e*). This is because higher temperature will promote the conversion of metastable zeolite into a more thermodynamically stable phase [30]. Thus, to obtain pure-phase Na-A zeolite from CFA and to lower the energy consumption for the synthesis, the crystallization temperature should be controlled in the range 80–100°C.

### 3.2. Hydrothermal synthesis of zeolite Na-X from coal fly ash

**Figure 4.**
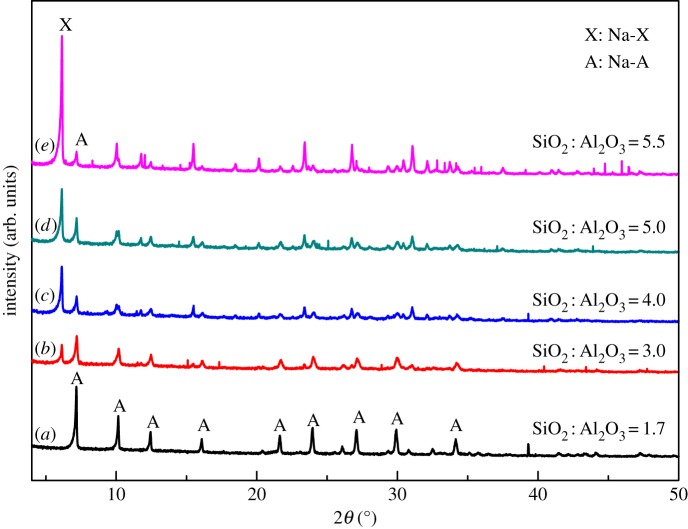
The impact of SiO_2_/Al_2_O_3_ molar ratio on the structure of the hydrothermal reaction products at 100°C for 8 h with additional silica sol and sodium hydroxide (Na-A: PDF 38-0241; Na-X: PDF 38-0237).

As aforementioned, the Si/Al ratio in the CFA sample used eventually satisfied the requirement for the synthesis of Na-A zeolite. It would be more interesting if other zeolites of industrial significance can be synthesized by altering the Si/Al ratio. We then investigated the potential synthesis of Na-X from the same CFA sample by changing the composition of calcinated Na_2_CO_3_/CFA mixture with silica sol and using NaOH to keep the Na/Si ratio to satisfy the requirements for Na-X crystallization. In the controlled experiments, silica sol and NaOH were added to the calcinated Na_2_CO_3_/CFA mixture to keep the Na_2_O/SiO_2_ and H_2_O/Na_2_O constant as 2.2 and 40, respectively. The duration for crystallization was 8 h and the crystallization temperature was 100°C above which SOD zeolite will appear in the product (figure 3). In this way, the SiO_2_/Al_2_O_3_ molar ratio in the reaction mixture is controlled to change from 1.7 to 5.5 upon introduction of silica sol and NaOH. The XRD patterns of the resulting products are shown in figure 4.

According to XRD patterns, phase-pure zeolite Na-A was obtained when the SiO_2_/Al_2_O_3_ molar ratio was 1.7 (figure 4*a*). The characteristic XRD peaks of zeolite Na-A disappear and new peaks associated with zeolite Na-X appear when the SiO_2_/Al_2_O_3_ molar ratio reaches 3 (figure 4*b*). With the increase of SiO_2_/Al_2_O_3_ molar ratio, the peaks of Na-X get intensified and sharpened and those of Na-A disappear completely (figure 4*c–e*). This is in excellent agreement with previous reports [30]. These findings prove that the SiO_2_/Al_2_O_3_ molar ratio is a key parameter to obtain phase-pure zeolite Na-X.

**Figure 5.**
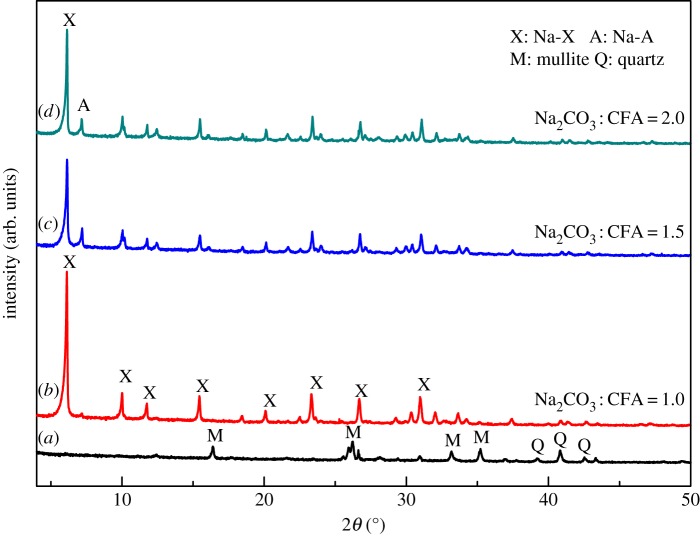
The effect of Na_2_CO_3_/CFA mass ratio on the structure of the products (mullite: PDF 83-1181; quartz: PDF 70-3755; Na-A: PDF 38-0241; Na-X: PDF 38-0237).

Alkalinity in the reaction mixture is another important parameter determining the crystallinity and composition of product, and is conventionally termed as Na/Si ratio. As calcinated Na_2_CO_3_/CFA mixture was used for the zeolite synthesis, we adapted the Na_2_CO_3_/CFA mass ratio to represent the Na/Si ratio in the reaction mixture before the hydrothermal crystallization. In controlled experiments, the Na_2_O/SiO_2_ and H_2_O/Na_2_O were kept constant as 2.2 and 40, respectively. The crystallization temperature was 100°C and the duration for crystallization was 8 h. The XRD patterns of the products after hydrothermal crystallization are shown in figure 5. When only tablet-compressed calcinated CFA was used as reactant (figure 5*a*), there are only quartz and mullite in the product, showing that Na_2_CO_3_ is required to convert quartz and mullite into precursors for zeolites. When the Na_2_CO_3_/CFA ratio is increased to 1, phase-pure Na-X zeolite was obtained, as indicated by the sharp characteristic XRD peaks of Na-X in figure 5*b*. When the Na_2_CO_3_/CFA ratio is increased further, the characteristic peaks of Na-A appear on the XRD patterns of the products (figure 5*c*,*d*). This shows that a mixture of Na-A and Na-X will be obtained at Na_2_CO_3_/CFA ratio larger than 1. Therefore, Na_2_CO_3_/CFA ratio is an important factor that governs the zeolite crystallization and the optimum Na_2_CO_3_/CFA ratio for the crystallization of Na-X was 1.0. According to the chemical composition of the CFA (table 1) and the aforementioned experiments, we concluded that phase-pure zeolite Na-A can be synthesized with CFA at reactant molar ratio, hydrothermal reaction temperature and reaction time of 1.3Na_2_O: 0.6Al_2_O_3_: 1SiO_2_: 38H_2_O at 80°C for 6 h, respectively, while phase-pure zeolite Na-X can be synthesized at 2.2Na_2_O: 0.2Al_2_O_3_: 1SiO_2_: 88H_2_O at 100°C for 8 h, respectively. With these set-ups, the yields of Na-A and Na-X are approximately 68% and approximately 62%, respectively. The relatively low yield of Na-X is due to the introduction of silica sol to get phase-pure Na-X [60].

### 3.3. Characterization of zeolite Na-A and Na-X

We then characterized the chemical composition of synthesized Na-A and Na-X samples with XRF. The result of XRF on Na-A sample indicates that it contains 44.64% SiO_2_, 37.29% Al_2_O_3_, 18.22% Na_2_O, 3.80% CaO, 3.36% Fe_2_O_3_, 3.06% TiO_2_, 0.55% MgO and 0.41% K_2_O and the corresponding Si/Al molar ratio is 1.48 and is typical for commercial Na-A, while that for the Na-X sample is 5.12 [30,61,62]. The high Si/Al molar ratio of Na-X is due to the introduction of silica sol to get phase-pure Na-X [60].

**Figure 6.**
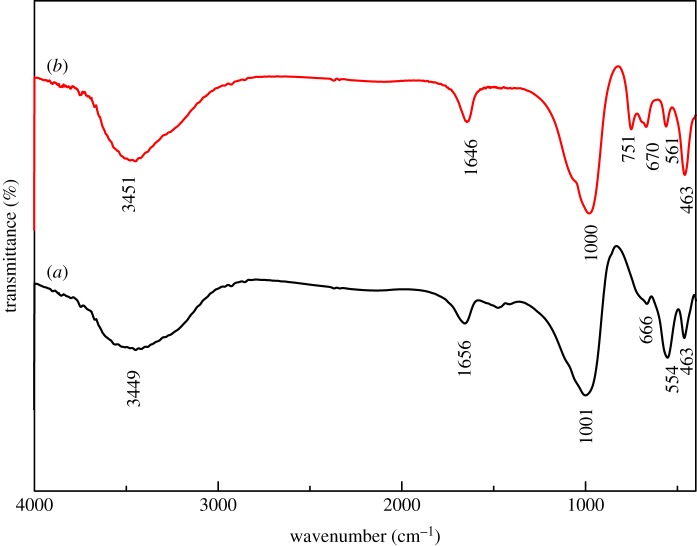
FT-IR spectra of Na-A (*a*) and Na-X (*b*) zeolites synthesized from calcinated CFA.

FT-IR spectroscopy was used to confirm the structure of synthesized Na-A and Na-X samples, as shown in figure 6. FT-IR bands at 463, 554, 666, 1001, 1656 and 3449 cm^−1^ were observed (figure 6*a*) for the Na-A sample. The band at 554 cm^−1^ was associated with the external vibration of double four-rings of Na-A zeolite framework. The band at 1001 cm^−1^ was related to the internal vibration of T-O asymmetric stretching. The band at 666 cm^−1^ was attributed to the internal vibration of T-O symmetric stretching. The band at 463 cm^−1^ was ascribed to the internal vibration of T-O bending. Moreover, the bands observed at 1656 and 3449 cm^−1^ correspond to the presence of H_2_O and hydroxyls, respectively. The observed FT-IR bands of Na-A samples are in good agreement with those reported in previous works, which further proves the successful synthesis of Na-A from CFA [30,31].

We also examined the FT-IR spectrum of the synthesized zeolite Na-X sample. As shown in figure 6*b*, Na-X zeolite has FT-IR bands at wavenumbers of 459, 561, 669, 747, 982, 1646 and 3451 cm^−1^. A broad band at 3451 cm^−1^ and the sharp peak at 1646 cm^−1^ can be assigned to the structural hydroxyl groups and bending mode of physically adsorbed water, respectively. The peak at 561 cm^−1^ can be attributed to the vibration of distorted double five-membered ring in the high silica ferrierite framework [63]. The bands at 1000, 751 and 670 cm^−1^ may be ascribed to the asymmetric and symmetric stretching vibration modes of Si–O tetrahedra. The peak at 463 cm^−1^ is attributed to the bending modes of T-O-T bridges. The observed FT-IR spectrum of Na-X zeolite in this study is also similar to those reported previously [42,64].

**Figure 7.**
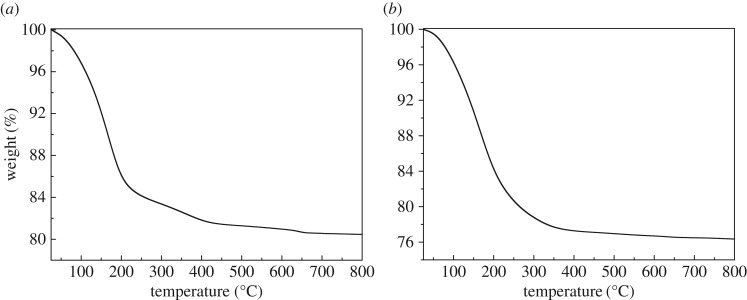
TGA of Na-A (*a*) and Na-X (*b*) zeolites synthesized from calcinated CFA.

TGA was also performed for Na-A and Na-X samples and the thermographs are shown in figure 7. Moisture loss from CFA-based zeolite Na-A starts between 40°C and 50°C and continues up to approximately 200°C (figure 7*a*). The observed percentage weight loss of moisture was approximately 20. The TGA thermograph of Na-X zeolite (figure 7*b*) indicates that desorption of physically adsorbed water molecules within the micropores was approximately 24%. The notable weight loss in the temperature range 40–200°C for zeolites Na-A and Na-X could be associated with the loss of free and physically adsorbed water inside the zeolite pores. The relatively large difference between moisture loss for the Na-A and Na-X observed in figure 7 could be explained with the hierarchical nature of the CFA-based zeolite Na-X that exhibits a greater external surface area. As shown in table 2, the surface area of synthesized Na-X sample was characterized by N_2_ adsorption (BET). The measured specific surface area is 573 m^2^ g^–1^, the single-point total pore volume is 0.2812 cm^3^ g^–1^ at a relative pressure (*p*/*p*_o_) of 0.198. These values are in good accordance with those reported by Sapawe *et al.* and Franus *et al*. [31,65]. The measured BET surface area of Na-X sample is bigger than that of Na-X synthesized with structure-directing reagents which will block some of the channels [66]. As the pore size of Na-A of 3--4 Å is much smaller than that of Na-X and is similar to the dynamic radii of N_2_, we did not measure the BET surface area of the synthesized Na-A sample.

**Table 2.** Zeolite pore volume and diameter.

zeolite	pore vol. (cm^3^ g^−1^)	average diameter (nm)	BET surface area (m^2^ g^−1^)
Na-X	0.2812	1.96	573.71

**Figure 8.**
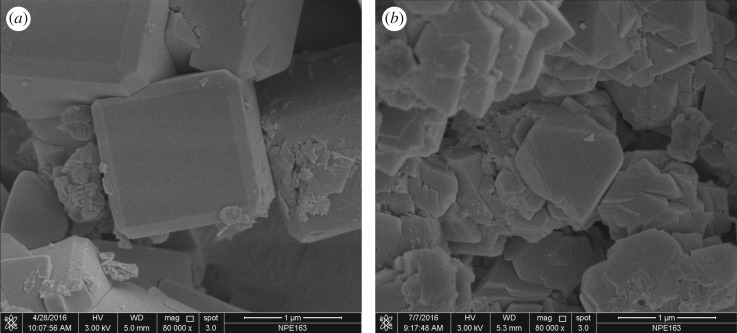
SEM images of synthesized Na-A (*a*) and Na-X (*b*) zeolites obtained from calcinated CFA.

The morphology of the synthesized Na-A and Na-X samples was examined and the corresponding SEM micrographs are shown in figure 8. The synthesized Na-A sample is of cubic morphology and is consistent with most of the reports on phase-pure zeolite Na-A. As for the Na-X samples, most of them are of rhombus morphology. The average crystal size, as estimated using the cumulative distribution method, ranges from 1 to 2 µm.

## 4. Conclusion

We showed that tablet compression can enhance the contact with Na_2_CO_3_ for the activation of CFA through calcination for the synthesis of zeolites Na-A and Na-X under mild conditions. We optimized the control variables for zeolite synthesis and showed that phase-pure zeolite Na-A can be synthesized with CTA at reactant molar ratio, hydrothermal reaction temperature and reaction time of 1.3Na_2_O: 0.6Al_2_O_3_: 1SiO_2_: 38H_2_O at 80°C for 6 h, respectively, while phase-pure zeolite Na-X can be synthesized at 2.2Na_2_O: 0.2Al_2_O_3_: 1SiO_2_: 88H_2_O at 100°C for 8 h, respectively. Further SEM, BET, FT-IR and TGA characterization confirmed that the Na-A and Na-X synthesized under optimized conditions would exhibit properties the same as phase-pure Na-A and Na-X. In this sense, the zeolites synthesized can principally be used as adsorbents for gas separation, wastewater treatment and soil remediation, as heterogeneous Lewis/Bronsted acid catalysts for the conversion of chemicals, as support materials for stabilization and dispersion of catalytic reaction centres, as cation-exchange materials for resource recovery and in other industrial applications where Na-A and Na-X play a role. Furthermore, the developed protocols for the synthesis of Na-A and Na-X from CFA are simple and cost- and energy-effective, so they can be adapted for mass production of zeolites in chemical plants. Related works are now being carried out in our laboratory.

## Data accessibility

Data are available from the Dryad Digital Repository (http://dx.doi.org/10.5061/dryad.s145h) [67].
